# Investigating the interplay between intrinsic and evoked activities in cultured neuronal networks by dimensional reduction techniques and high-density MEAs

**DOI:** 10.1186/1471-2202-14-S1-P24

**Published:** 2013-07-08

**Authors:** Thierry Nieus, Alessandro Maccione, Luca Berdondini

**Affiliations:** 1 Istituto Italiano di Tecnologia (IIT), Neuroscience and Brain Technology Department - Genoa, Italy

## 

High density microelectrode arrays (MEAs) provide extracellular recordings from thousand of electrodes (http://www.3brain.com) and offer novel capabilities to investigate electrophysiological signaling in cultured neuronal networks and in *ex vivo *brain tissues. In this study we report on our recent technological and data analysis advancements to investigate the propagation and the interplay of spontaneous and electrically evoked activities in cultured networks. To do so, a novel high density MEA with on-chip stimulating electrodes was realized. It provides whole-array recordings from 4096 electrodes (pitch of 81 um, active area of 8 mm by 8 mm) and electrical stimulation from 16 electrodes located every 8 recording sites. Here, this device was used to interface hippocampal neuronal networks. From the second week in vitro [1] these cultures display a peculiar intrinsic firing regime characterized by periodic synchronized network-wide bursts. These network bursts originate from specific ignition sites of the cultured network (i.e. characterized by more excitable cells) and can propagate through the entire network. These propagations are informative of the underlying network connectivity and their classification based on their spatiotemporal patterns might elucidate the network's organization and its ongoing dynamic. Previous studies [1] have described the trajectories of these propagations by tracking their center of activity trajectory (CAT). Although CATs provide a good overall description of spatiotemporal patterns, they are not suited for fine studies on these propagations. Here we have adopted a more rigorous approach by applying dimensional reduction techniques that take advantage of the redundancy and of the sparseness of multi-unit recordings. Our results show that by *Principal Component Analysis *(PCA) we can properly reconstruct the time course of spatiotemporal propagations with a minimal set of three components (i.e. explaining ~90% of the variance of the trajectories). The PCA classification based approach allowed us to characterize both intrinsic and electrically evoked network-wide propagations. Interestingly, our results show that electrical stimulation can evoke (i) distinctive propagations that depend on the specific spatial-temporal properties of the stimulus as well as (ii) propagations that are already expressed in the intrinsic 'repertoire' of network bursts. Here we will discuss these results and our observations on the interplay between intrinsic and electrically evoked network-wide propagations.

## References

[B1] Gandolfo M Maccione A Tedesco M Martinoia S Berdondini L Tracking burst patterns in hippocampal cultures with high-density CMOS-MEAs J Neural Eng 2010 7 5 Oct 10.1088/1741-2560/7/5/056001 20720282

